# Stable ICG-loaded upconversion nanoparticles: silica core/shell theranostic nanoplatform for dual-modal upconversion and photoacoustic imaging together with photothermal therapy

**DOI:** 10.1038/s41598-017-16016-x

**Published:** 2017-11-16

**Authors:** Ruichan Lv, Depeng Wang, Liyang Xiao, Guanying Chen, Jun Xia, Paras N. Prasad

**Affiliations:** 1 Institute for Lasers, Photonics, and Biophotonics and Department of Chemistry, University at Buffalo, the State University of New York, Buffalo, NY 14260 USA; 2 Department of Biomedical Engineering, University at Buffalo, the State University of New York, Buffalo, NY 14260 USA; 3 Engineering Research Center of Molecular and Neuro Imaging, Ministry of Education, School of Life Science and Technology, Xidian University, Xi’an, Shanxi 710071 China

## Abstract

We report here the design and multiple functions of a new hierarchical nanotheronostic platform consisting of an upconversion nanoparticle (UCNP) core: shell with an additional mesoporous silica (mSiO_2_) matrix load shell containing sealed, high concentration of ICG molecules. We demonstrate that this UCNP@mSiO_2_-ICG nanoplatform can perform the following multiple functions under NIR excitation at 800 nm: 1) Light harvesting by the UCNP shell containing Nd and subsequent energy transfer to Er in the Core to produce efficient green and red upconversion luminescence for optical imaging; 2) Efficient nonradiative relaxation and local heating produced by concentration quenching in aggregated ICG imbedded in the mesopourous silica shell to enable both photoacoustic imaging and photothermal therapy. Compared to pure ICG, sealing of mesoporous silica platforms prevents the leak-out and improves the stability of ICG by protecting from rapid hydrolysis. Under 800 nm laser excitation, we performed both optical and photoacoustic (PA) imaging *in vitro* and *in vivo*. Our results demonstrated that UCNP@mSiO_2_-ICG with sealed structures could be systemically delivered to brain vessels, with a long circulation time. In addition, these nanoplatforms were capable of producing strong hyperthermia efforts to kill cancer cells and hela cells under 800 nm laser irradiation.

## Introduction

In the field of biomedical imaging, there is an emerging need for multi-modal probes that can incorporate different imaging/therapeutic modalities to provide comprehensive information about the tissue, and to offer precise and targeted therapy^1–6^. While conventional nuclear imaging modalities, such as X-ray computed tomography and positron-emission tomography, have played a critical role in preclinical and clinical imaging, there are concerns about ionizing radiation which may cause cancer. Alternatively, optical imaging modalities, such as fluorescence imaging, can reveal functional and molecular information about the tissue using “safe” visible or near infrared light. In the fluorescence imaging field, upconversion nanoparticles (UCNPs) have been proposed as an effective optical tool which could transfer the near-infrared light to visible emission using the ladder-like energy levels of rare-earth ions^7–9^. UCNPs show advantages such as, high physical/chemical stability, good biocompatibility, non-blinking, absence of the background signal, and high sensitivity^10–12^. Attempts have been made in the past years to combine UCNPs with other photo-active agents such as organic dyes and amphiphilic polymers through physical co-encapsulation and/or chemical link^13–15^. However, due to optical diffusion, most optical imaging techniques quickly loss spatial resolution beyond a few millimeters of penetration depth. Such a superficial penetration depth could not be satisfactory for clinical imaging of solid tumors of brain^16–23^.

If several imaging modalities could be integrated, the multi-modal imaging agents could be potential imaging agents for clinical application. These agents could combine the typical features of optical imaging and other imaging (magnetic resonance imaging, computed tomography, etc)^24–26^. Recently, there are increasing interests in photoacoustic (PA) imaging, which allows for deep-tissue image of optical absorption at ultrasound-defined spatial resolutions (100–300 µm resolution for a 5 MHz transducer)^27–30^. Over the past few years, numerous contrast agents have been developed for PA imaging. However, most of them work purely as a signal enhancement medium and do not offer other imaging or therapeutic capacities. In this study, we developed a novel nanoplatform that enable both PA and up-converting luminescence imaging, and can stably encapsulate chemical dyes or molecules for therapeutic applications. Promisingly, the combination of upconversion luminescence with PA imaging would expand the penetration depth of other biomedical imaging or therapy tools. For instance, visible luminescence from UCNPs upon NIR irradiation can be used to excite visible-light-sensitive photosensitization in deep tissue^31,32^, while photoacoustic imaging could help locate UCNPs to guide the photodynamic therapy.

The PA contrast of our platform originates mainly from indocyanine green (ICG), which is the only FDA-approved chemical dye that strongly absorbs 740–800 nm wavelength lights^33–35^. Due to the weak tissue absorption and scattering, near-infrared (NIR) lights are well suited for clinical imaging applications. However, ICG has several major drawbacks, such as poor stability in aqueous solution, temperature and light dependent optical features, and rapid blood clearance, all of which restrain its applications in the long run^36–38^. Strategies like drug delivery systems have been proposed to address these intrinsic issues^37–40^. In particular, mesoporous silica platforms have been considered as one of the most promising systems because of their unique advantages, including high pore volume, uniform pore size, and high Brunauer, Emmett and Teller (BET) surface area^41–44^. However, in these systems, it is still challenging to find an effective way to prevent the leak-out and to improve the stability of the drug molecules.

Herein, core/shell mesoporous matrix was synthesized with a sealed TEOS-mediated structure, which could effectively load adjustable amount of ICG molecules and protect it from rapid hydrolysis. To evaluate the long-term stability, we compared the absorbance spectra of ICG in the sealed structure and in its original format after both have been kept for several days. We also demonstrated the dual-modal imaging capability of the sealed structure *in vivo* and *in vitro* using 800 nm light as the excitation source. Moreover, we tested the hyperthermia property of the system and confirmed that it could produce sufficient hyperthermia to kill cancer cells under 800 nm laser irradiation.

## Results

**Figure 1:**
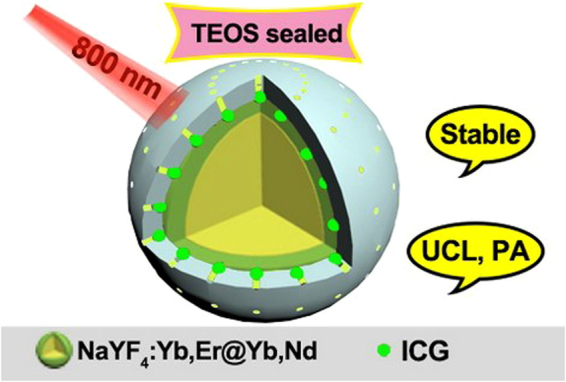
The Schematic diagram of the fabricated sable nanoparticles for UCL and photoacoustic imaging.

**Figure 2:**
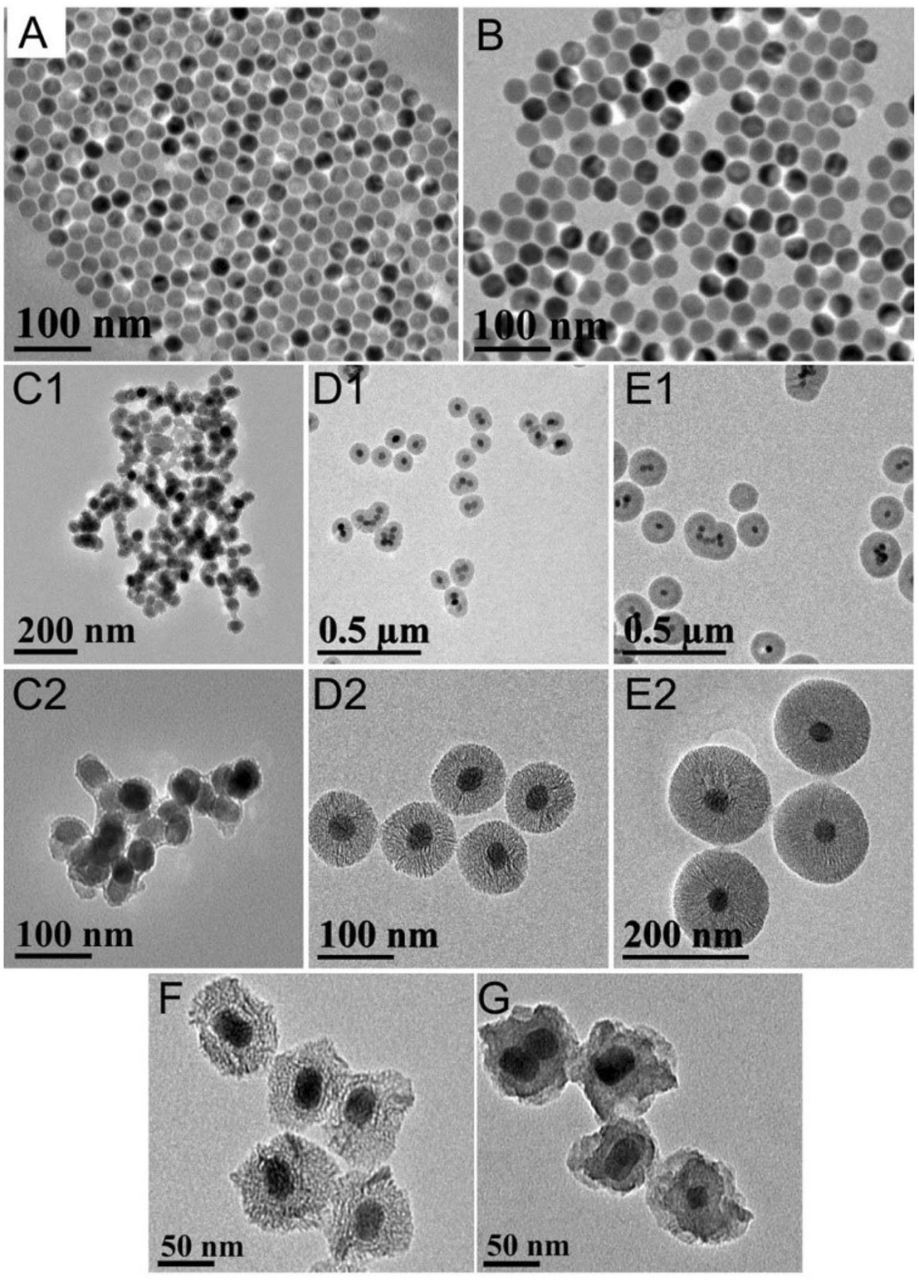
TEM images of (**A**) NaYF_4_:18%Yb,2%Er and (**B**) NaYF_4_:18%Yb,2%Er@NaYF_4_:30%Nd,10%Yb UCNPs. TEM images of core/shell UCNP@mSiO_2_ with different sizes (**C1**,**C2**) 5 nm, (**D1**,**D2**) 30 nm, and (**E1**,**E2**) 60 nm. TEM images of UCNP@mSiO_2_-ICG with (**F**) open structure and (**G**) sealed structure.

Figure 1 shows the schematic diagram of our new core/shell nanoparticles with TEOS sealed. The stable UCNP@mSiO_2_-ICG with sealed structure can produce upconversion luminescence (UCL) and PA imaging simultaneously, as well as perform photothermal therapy. TEM images of the core and the core/shell nanoparticles are shown in Fig. 2A,B, indicating that the samples are uniform. The size of the NaYF_4_:18%Yb,2%Er core is ~25 nm, and it increases to ~32 nm after coating with a NaYF_4_:30%Nd,10%Yb shell. We found that a good morphology is essential for further coating with mesoporous silica. With different amount of TEOS added, the corresponding added mesoporous silica thickness of UCNP@mSiO_2_ is 5 nm (Fig. 2C1,C2), 30 nm (Fig. 2D1,D2), and 60 nm (Fig. 2E1,E2), respectively. UCNP@mSiO_2_ with silica size of 30 nm was taken as the typical structure and sealed with TEOS. Compared to the open mesoporous structure (Fig. 2F), the UCNP@mSiO_2_ with ICG loaded and completely sealed has evidently decreased mesopores and channels (Fig. 2G).

**Figure 3:**
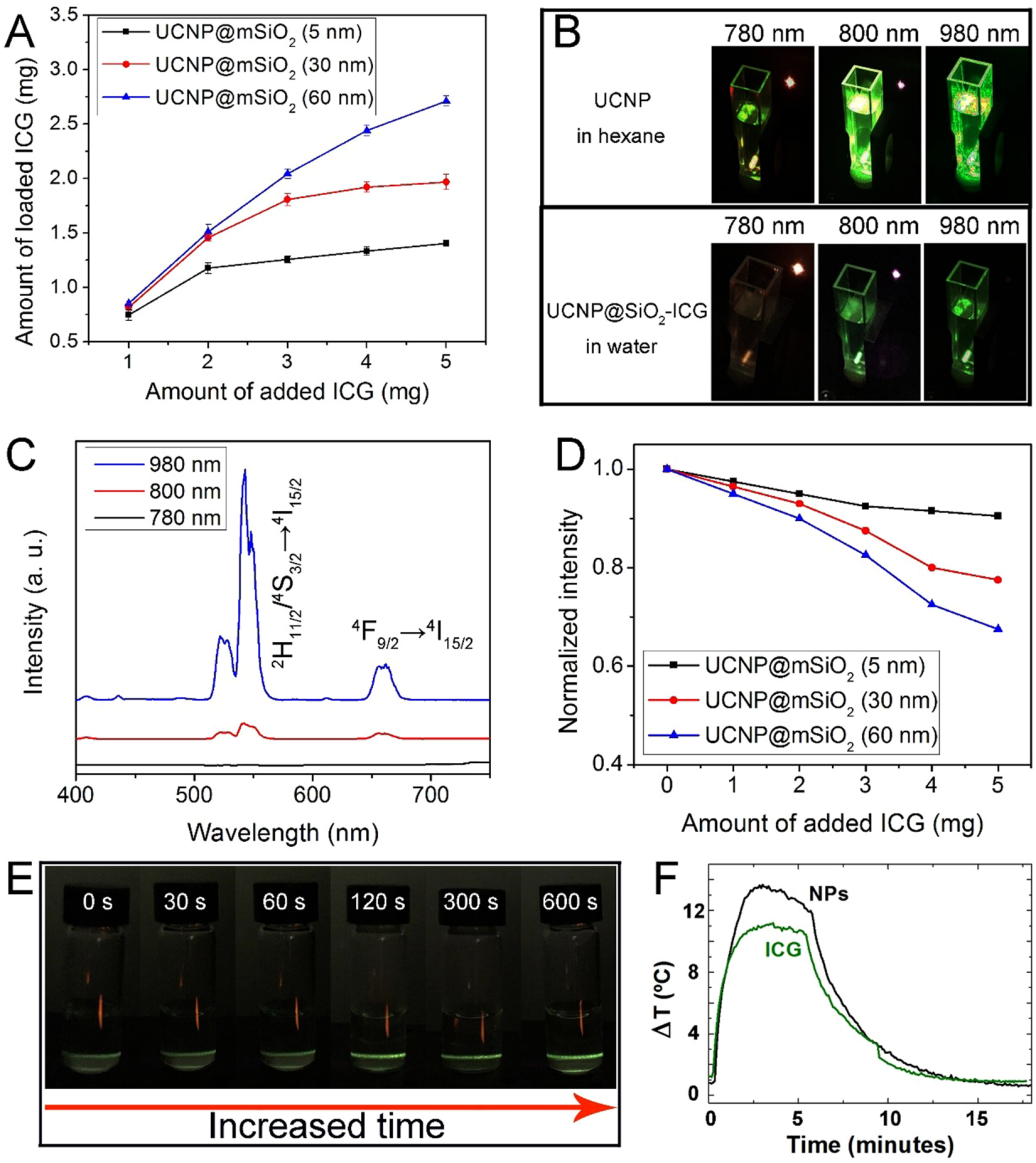
(**A**) The amount of loaded ICG versus the added ICG molecules. (**B**) The photographs of UCNPs in hexane and UCNP@mSiO_2_-ICG (30 nm) in water under different irradiation lasers. (**C**) The UCL spectra of UCNP@mSiO_2_-ICG (30 nm) in water under different irradiation lasers. (**D**) The normalized UCL intensity of UCNP@mSiO_2_-ICG (with different silica thickness of 5 nm, 30 nm, and 60 nm) with the increased amount of added ICG. (**E**) The photographs of UCNP@mSiO_2_-ICG (30 nm) in water with prolonging irradiation time. All of the pump powers are 1.02 W cm^−2^. (**F**) The temperature curves of UCNP@mSiO_2_-ICG and pure ICG under 800 nm irradiation.

Figure 3A shows the loaded amount of ICG for the aforementioned three samples (mesoporous silica thickness of UCNP@mSiO_2_ is 5 nm, 30 nm and 60 nm), which exhibit similar trends: the loaded amount of ICG molecules increases with increased added amount. Meanwhile, the maximum loaded amount increases when the silica thickness changed from 5 nm to 30 nm and further to 60 nm. Additionally, the experiment on the loading and leaking of ICG from sealed and non-sealed nanoparticles have been carried out (Figure S1). We took a total of 2.5 mg of ICG loading into 20 mg of UCNP@SiO_2_, and the final loading amount of ICG molecules to the sealed and non-sealed UCNP@SiO_2_ are 1.55 mg and 1.24 mg, respectively. Then, we measured the release properties within 12 h, the final leaking percent of the non-sealed UCNP@SiO_2_ and sealed UCNP@SiO_2_ are 62.9% and 15.1%, respectively. The upconversion efficiency under various excitation wavelengths is also stronger, when UCNP@mSiO_2_-ICG is diluted in hexane than those diluted in water (Fig. 3B). The emission spectra of UCNP@mSiO_2_-ICG (30 nm) are shown in Fig. 3C. Two main emission peaks in the green region (543 nm) and red region (650 nm) (for either 800 nm or 980 nm irradiations) are corresponding to ^2^H_11/2_/^4^S_3/2_ → ^4^I_15/2_ and ^4^F_9/2_ → ^4^I_15/2_ energy transfer process, respectively. Also, we detected the influence of the amount of added ICG to the UCL intensity. The intensity of the UCL emission decreased significantly after being loaded with ICG (Fig. 3D). This observation is contrary to the phenomenon we reported in^45^, where ICG was used to sensitize lanthanide ions through widening the irradiation region (absorbance area), which subsequently enhances the upconverting luminescence. The lack of luminescence enhancement in the current study indicates that in the aqueous environment, ICG molecules photo-quenched UCNP@mSiO_2_. As shown in Fig. 3E, once the aqueous UCNP@mSiO_2_-ICG (30 nm) has been irradiated for a long time, the upconversion emission gradually increased due to the decreased photo-quenching of ICG (photobleaching of the dye under irradiation). When the pump power density of 808 nm laser was 0.74 W/cm^2^, the corresponding maximum penetration depths of UCNP@mSiO_2_-ICG were 2 mm. Meanwhile, under 800 nm irradiation, the temperature increase of UCNP@mSiO_2_-ICG over pure ICG indicates that there is increased photothermal effect when ICG molecules are loaded in the mesoporous structure (Fig. 3F). This photothermal efficiency increases with an increase in the amount of loaded ICG, supporting that heating is produced by nonradiative relaxation derived from concentration quenching.

**Figure 4:**
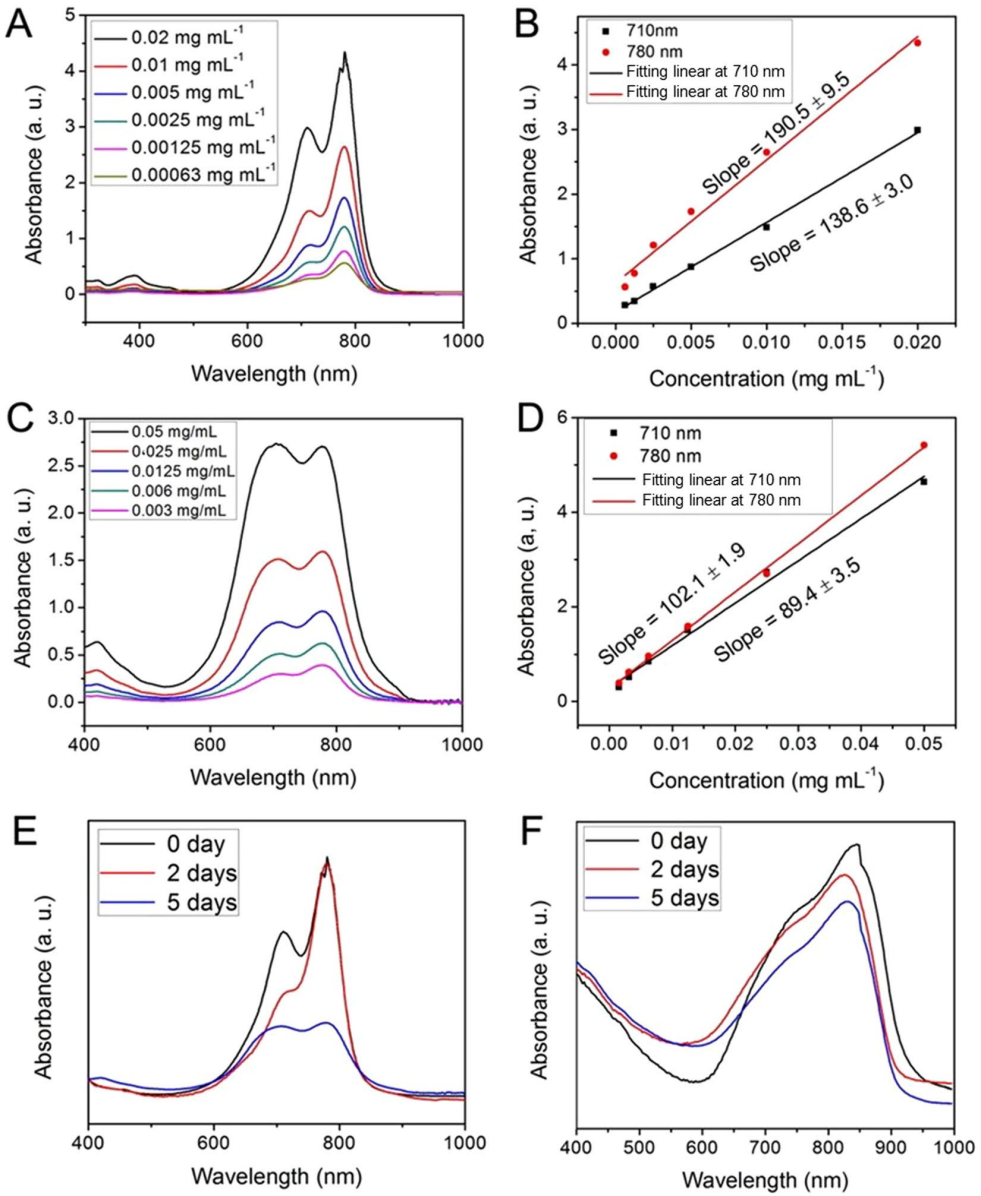
The UV-vis spectra and corresponding linear relation versus concentration of pure ICG dissolved into deionized water solution detected (**A**,**B**) immediately and (**C**,**D**) after being kept for days. UV-vis-NIR absorbance spectra of (**E**) pure ICG and (**F**) UCNP@mSiO_2_-ICG with sealed structure at different store time points. All the samples are dissolved into deionized water solution.

To investigate the stability, we also measured the absorption spectra of pure ICG and UCNP@mSiO_2_-ICG over time. As shown in Fig. 4A, fresh-made pure ICG exhibits two main absorption peaks at 710 nm and 780 nm, respectively. After being kept for several days, the absorbance of pure ICG changed dramatically (Fig. 4C). As shown in Fig. 4B and D, the absorbance versus concentration slope at 780 nm decreased from 190.5 to 102.1, and the slope at 710 nm decreased from 138.6 to 89.4. These changes indicate the instability of pure ICG in water after being kept for several days. In contrast, the absorbance of UCNP@mSiO_2_-ICG with the sealed structure can be retained over a long period of time without obvious decrease (Fig. 4E). Apparently, the encapsulation strategy protects ICG molecules inside mesoporous channels and pores, preventing degradation seen in the ambient aqueous solution.

**Figure 5:**
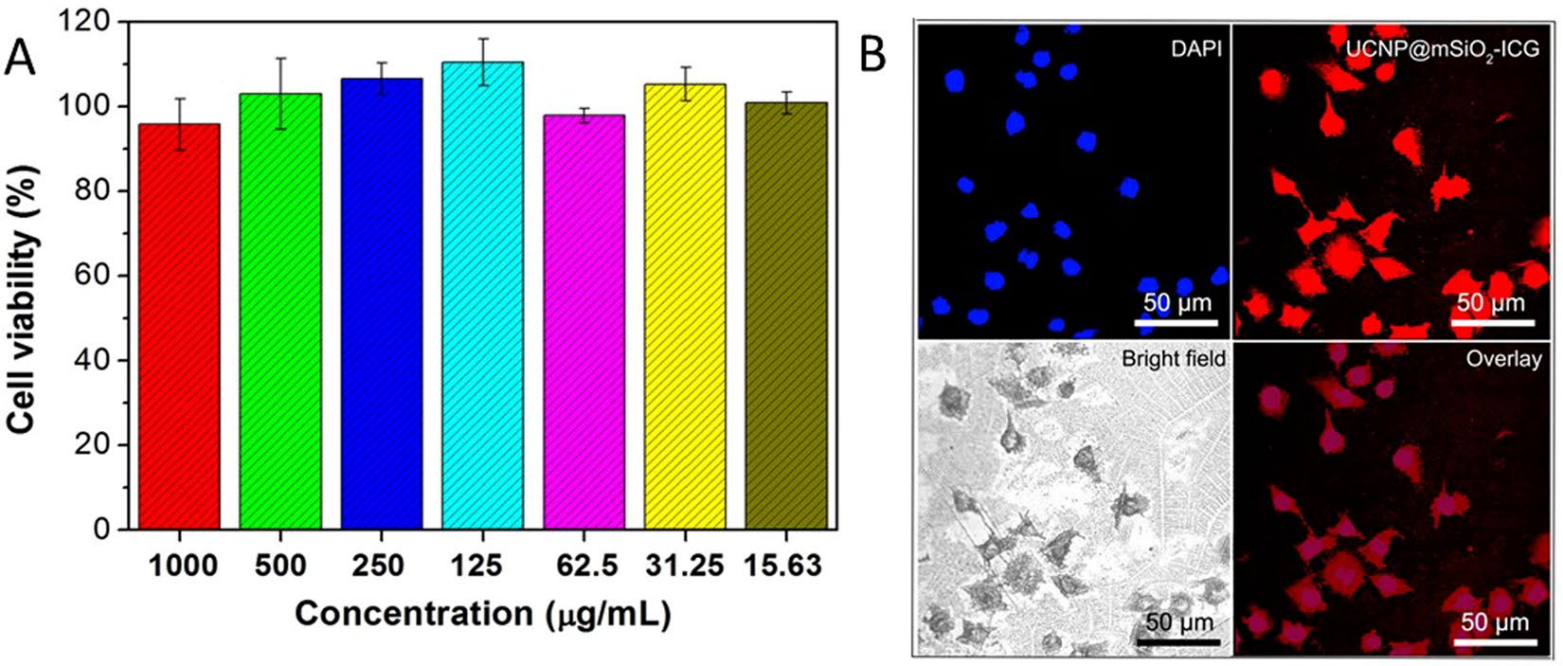
Images for cell experiment. (**A**) MTT assay using L929 cell lines incubated with UCNP@mSiO_2_-ICG with different concentrations. (**B**) Optical microscopy images of L929 cells that have been incubated with UCNP@mSiO_2_-ICG for 24 h.

To realize the *in vitro* imaging and therapeutic effect of UCNP@mSiO_2_-ICG, the biocompatibility of the as-synthesized sample was carried. As shown in Fig. 5, the viability of L929 cells incubated with UCNP@mSiO_2_-ICG with different concentrations was 98.5–109.8%. Moreover, the optical microscopy images of L929 cells incubated with UCNP@mSiO_2_-ICG for 24 h shows that there were almost no dead cells. These results indicate good biocompatibility of the as-synthesized samples.

**Figure 6:**
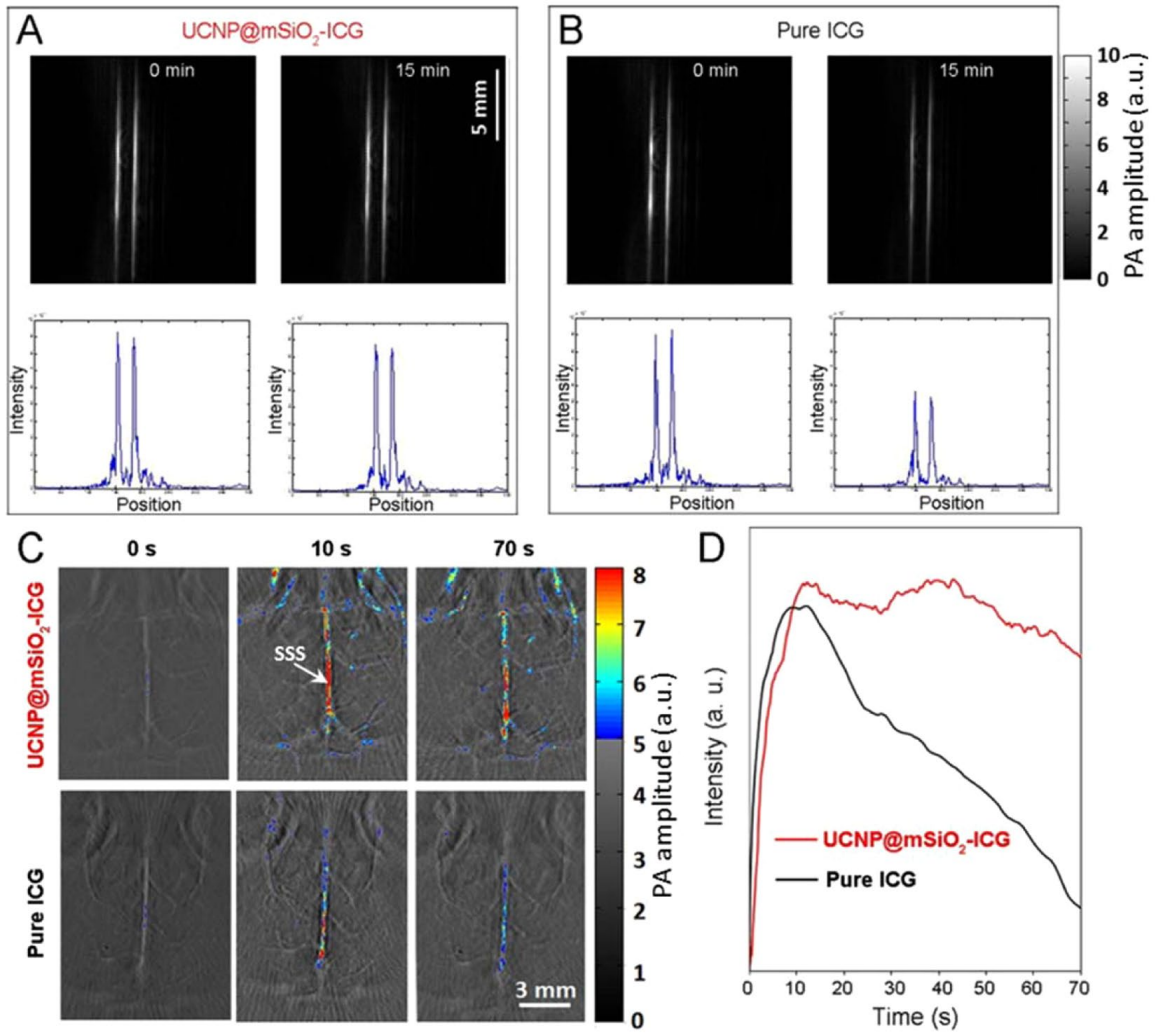
The photoacoustic imaging and corresponding signal intensity of (**A**) UCNP@mSiO_2_-ICG and (**B**) pure ICG under 800 nm laser with irradiation time at 0 min and 15 min. Note that the absorbance is normalized in 800 nm. (**C**) Mice brain imaging after injection of UCNP@mSiO_2_-ICG and pure ICG. The excitation wavelength is 800 nm with pulsed and continuous laer, n=3. SSS, superior sagittal sinus. (**D**) The *in vivo* photoacoustic intensity verusus the intravenous injection time.

PA images of UCNP@mSiO_2_-ICG and pure ICG are shown in Fig. 6A and B, respectively. Immediately after sample injection, the two tubes exhibit the same maximum signal intensity. However, after 15 min of irradiation with contineous wave (CW) laser, the PA amplitude of pure ICG decreased 40%, while that of UCNP@mSiO_2_-ICG with sealed structure only decreased 7%. We also verified the enhanced stability of the UCNP@mSiO_2_-ICG *in vivo*. Figure 6C top and bottom rows show *in vivo* PA brain images acquired after injection of UCNP@mSiO_2_-ICG and pure ICG, respectively. Immediately and 10 seconds after injection, both brains show similar PA signal amplitude. However, at 70 second, the mouse with UCNP@mSiO_2_-ICG injection exhibits a much higher PA signal in main cortex vessels than the one with pure ICG injection, owing to the enhanced stability and longer residence time of UCNP@mSiO_2_-ICG. A threshold was applied when we plotted all the images: pixels with intensity below the threshold were plotted in gray scale, while ones above the threshold were plotted in color. Figure 6D shows temporal changes of the PA amplitude in a main cortex vessel [marked as SSS (superior sagittal sinus) in Fig. 6C]. It can be seen that PA signals in the main vessel increased immediately after either the UCNP@mSiO_2_-ICG or ICG injection, and they reached the peak values at around 10 seconds. Following that, PA signals in both brains started to decrease at different rates. The one injected with UCNP@mSiO_2_-ICG exhibited much slower rate of decrease than the one injected with ICG. This experiment clearly demonstrates the enhanced stability of UCNP@mSiO_2_-ICG *in vivo*.

**Figure 7:**
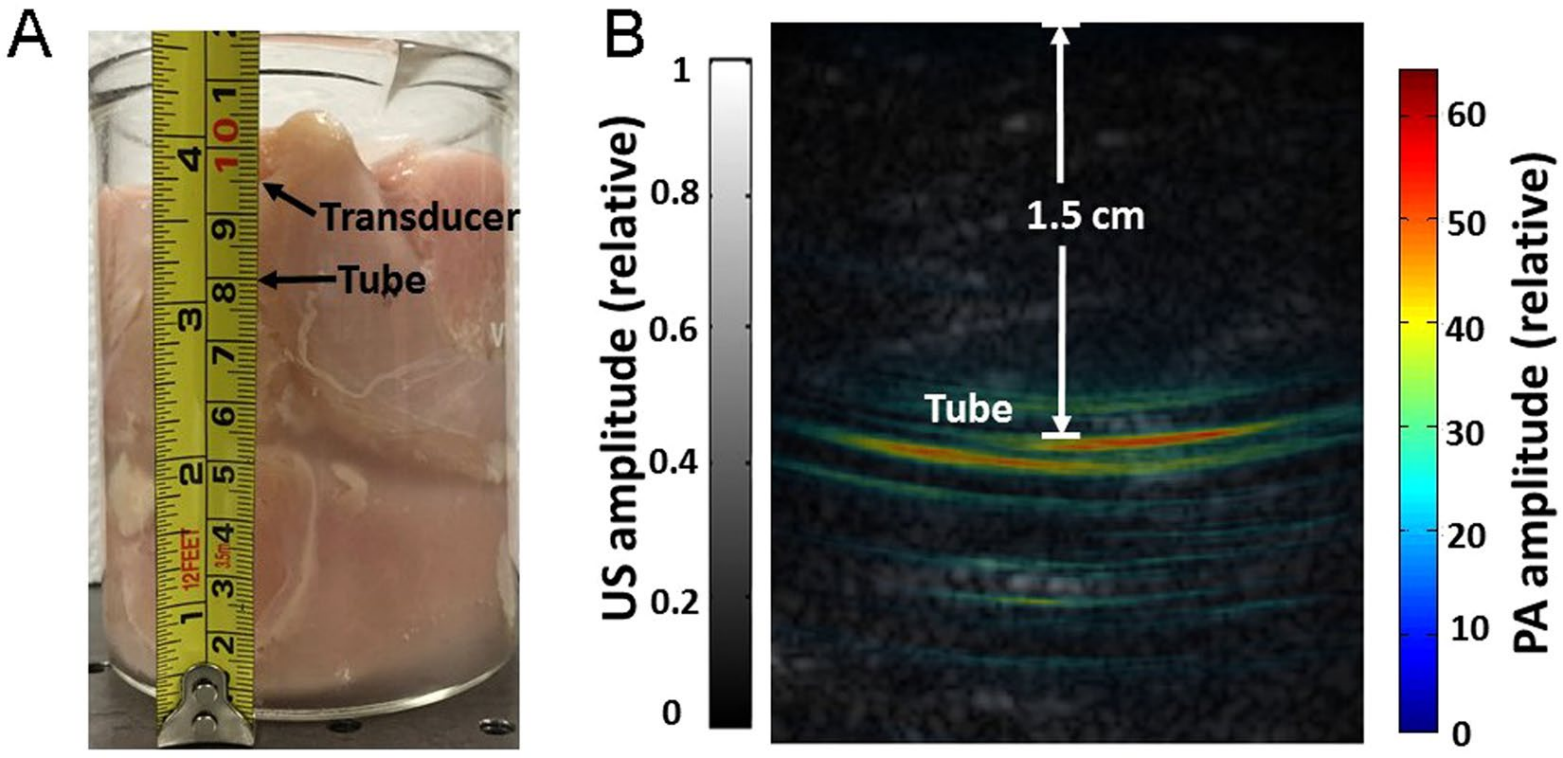
Detection of UCNP@mSiO_2_-ICG through 1.5 cm of tissue. (**A**) Photograph of the UCNP@mSiO_2_-ICG sample with chicken breast covered. (**B**) Overlaid photoacoustic (color) and ultrasound (gray) images.

To quantify the imaging depth, we gradually stacked chicken breast tissues on the top of the tube filled with UCNP@mSiO_2_-ICG (sample has been kept for 5 days) (Fig. 7A) and monitored the PA signal in real time. As the tissue thickness increased, the tube’s signal decreased and was eventually buried in background noise (without averaging), when the depth reached approximately 1.5 cm (measured with a ruler). We then stopped stacking chicken breast tissue and considered this distance as the deepest detection depth. One hundred frames were acquired at this depth, and all data were averaged to improve signal to noise ratio (SNR). The corresponding overlaid PA (color scale) and ultrasound (gray scale) images are shown in Fig. 7B, in which the tube is clearly visible with 16 dB SNR. The distance from tube to the transducer surface was calculated to be 1.5 cm. Even though the imaging depth is not as deep as those in previous studies^46,47^, the concentration of ICG (lower than 10/100 w/w) used in our study is lower than clinically approved concentration of ICG^48^, causing negligible side effects to cell (Fig. 5). We also imaged a tube filled with pure ICG (sample also has been kept for 5 days) through 1.5 cm chicken breast tissue. However, the tube cannot be detected due to fast photobleaching during those 5 days.

The inverted fluorescence microscope images of HeLa cells incubated with UCNP@mSiO_2_-ICG upon 808 nm NIR light irradiation were obtained. As shown in Figure S2, under the 808 nm irradiation, the cells incubated with the UCNP@mSiO_2_-ICG emit green light. We also demonstrated upconversion luminescence through subcutaneous injection of (UCNP@mSiO_2_-ICG). Under 500 mW 800 nm irradiation, we can clearly observe a green light through mouse skin (Figure S3), indicating the potential of dual-modal PA and upconversion luminescence imaging.

To evaluate the photothermal therapy effect, we used confocal laser scanning microscopy to image Hela cells incubated with nanoparticles. The live/dead assay was conducted for HeLa cells incubated with UCNP@mSiO_2_-ICG, and then irradiated with 808 nm light (10 min, 1.0 W/cm^2^). The scheme of photothermal experiment and the final confocal laser scanning microscopy image are presented in Figure S4. The cells were marked with calcein AM (dyed the live cells into green) and PI (dyed the dead cells into red). Hela cells were incubated with the culture and UCNP@mSiO_2_-ICG for 6 h, and then the center of the well was irradiated by the 808 nm laser. It can be seen that when the nanoparticle-incubated HeLa cells were exposed to 808 nm irradiation, nearly all the cancer cells were killed, implying the use of UCNP@mSiO_2_-ICG as the photothermal agent for anti-cancer therapy. For future clinical application, we also verified the bio-distribution. As shown in Figure S5, the nanoparticles mainly accumulated in the liver shortly after intravenous administration, and were mostly cleared out after 3 days. The photothermal effect of this platform were further proved through *in vivo* experiments. Tumors were established by subcutaneous injection of H22 cells in the left axilla of mice (18–22 g). One week after subcutaneous injection, tumor-bearing mice were separated into two groups: one is the control group without any treatments, while the other is the photothermal group with UCNP@mSiO_2_-ICG injected into the tumor, which was then irradiated with 808 nm light for 10 min at 1.0 W/cm^2^. Body weight and tumor size of mice were monitored every 2 days after treatment. Fourteen days post treatment, the body weight of both groups increased constantly (Figure S6A), indicating that nanoparticles have no obvious side effects to mice. Meanwhile, as shown in the inset of Figure S6A, there were obvious temperature increase at the tumor site during photothermal treatment. Figure S6B shows changes in tumor size for both groups. Tumor size and growth rate of the photothermal group are significantly smaller than those of the control group. This *in vivo* result further proves that our nanoparticle platform can potentially be used as a theranostic agent.

## Discussion

In summary, we introduced a core/shell/shell matrix with amesopore-sealed structure, which could effectively load various amount of ICG molecules and protect it from rapid hydrolysis. Unlike pure ICG, the sealed structure can be kept for many days without significant changes in absorbance. With 800 nm laser, *in vitro* and *in vivo* PA imaging experiments demonstrated that UCNP@mSiO_2_-ICG could be delivered to brain vessels with better angiography depth and longer circulation time. Upconversion luminescence can also be seen after subcutaneous injection of nanoparticles. In addition, this nanoplatform was capable of producing sufficient hyperthermia to kill cancer cells under 800 nm NIR irradiation. These studies indicate that our new nanoplatform holds great potential for theranostic applications.

## Methods

### Reagents and Materials

Yttrium(III) oxide (Y_2_O_3_, 99.99%), ytterbium (III) oxide (Yb_2_O_3_, 99.9%), erbium (III) oxide (Er_2_O_3_, 99.99%), neodymium (III) oxide (Nd_2_O_3_, 99.99%), yttrium (III) acetate hydrate (Y(CH_3_COO)_3_, 99.99%), ytterbium (III) acetate tetrahydrate (Yb(CH_3_COO)_3_•4H_2_O, 99.99%), neodymium (III) acetate hydrate (Nd(CH_3_COO)_3_, 99.99%), trifluoroacetic acid (CF_3_COOH, 99%), sodium trifluoroacetate (CF_3_COONa, 98%), oleic acid (90%, tech grade) and octadecene (90%, tech grade), Indocyanine green, ammonia fluoride (NH_4_F, ≥99.99% trace metals basis), sodium hydroxide (NaOH, anhydrous, ≥97%) were obtained from Sigma Aldrich. Methanol (ACS reagent grade, ≥99.8%) and hexane (ACS reagent grade, ≥98.5%) were purchased from Fisher Scientific. All chemical reagents were used as received without any further purification.

### Synthesis of NaYF4:18%Yb,2%Er

Typically, 0.8 mmol of Y(CH_3_COO)_3_, 0.18 mmol of Yb(CH_3_COO)_3_, and 0.02 mmol of Er(CH_3_COO)_3_ were mixed in the three-necked bottle with 10 mL of oleic acid and 15 mL of octadecene. The mixture was heated to 160 °C under the argon atmosphere and kept at this termperature for 1 h, yielding a clear yellow solution (Solution A). Meanwhile, 4 mmol NH_4_F and 2.5 mmol of NaOH were mixed and sonicated for 10 min to obtain a clear solution (Solution B). After cooling Solution A down to the room temperature, Solution B was loaded into Solution A. After stirring for 30 mins, the mixed solution was heated to 100 °C for 20 min, followed by heating to 300 °C and kept for 1 h. After cooling down to room temperature, the solution was washed with ethanol and centrifuged to obtain NaYF_4_:18%Yb,2%Er nanoparticles. The collected nanoparticles were finally dispersed in hexane for further uses.

### Synthesis of core/shell NaYF_4_:18%Yb,2%Er@NaYF_4_:30%Nd,10%Yb

First, 0.6 mmol of Y_2_O_3_, 0.3 mmol of Nd_2_O_3_, and 0.1 mmol of Yb_2_O_3_ were mixed with 5 mL of CF_3_COOH and heated at 120 °C in a three-neck bottle to prepare corresponding lanthanide trifluoroacetate. Subsequently, hexane-dispersed NaYF_4_:18%Yb,2%Er core (1 mmol), 10 mL of oleic acid, and 15 mL of octadecene were loaded and heated at 120 °C for 30 mins under the argon atmosphere. Then, the mixture was heated to 310 °C and kept at this temperature for 30 mins. Finally, the solution was naturally cooled down to room temperature, and the core/shell nanoparticles were precipitated with ethanol, followed by washing with ethanol several times. After dried in the air, the NaYF_4_:18%Yb,2%Er@NaYF_4_:30%Yb,10%Nd (noted as UCNP) was finally dispersed in hexane for future uses.

### Synthesis of mesoporous UCNP@mSiO_2_

The oleic-UCNP was firstly converted to hydrophilic UCNP by ligand exchange with CTAB. Typically, 2 mL of NaYF_4_:18%Yb,2%Er@NaYF_4_:30%Yb,10%Nd and 0.1 g of CTAB were mixed with 10 mL ethanol and 40 mL of deionized water under continuous stirring. After stirring for 12 h, the solution turned clear without surplus hexane. Then, 1 mL of ammonia water was added. The mixture was heated and kept at 70 °C, and 150 μL of TEOS was added slowly. After keeping for 10 min, the mixture was centrifuged and washed several times with ethanol and water. The samples with different silica thicknesses were synthesized by adjusting the added amount of TEOS (0.15 mL, 0.20 mL, and 0.30 mL).

To remove the CTAB surfactant, the as-synthesized silica-coated spheres were mixed with 30 mL of ethanol with 0.3 g of NH_4_NO_3_, and then kept at 60 °C for 2 h. Then, the solution was centrifuged with ethanol three times and the precipitate was collected and denoted as UCNP@mSiO_2_.

### Synthesis of UCNP@mSiO_2_ –ICG with sealed structure

UCNP@mSiO_2_ with the open structure was firstly loaded with ICG through physical encapsulating. Typically, UCNP@mSiO_2_ was dispersed into 20 mL of deionized water and then mixed with different amounts (1, 2, 3, 4, and 5 mg) of ICG molecules. The solution was centrifuged and washed with deionized water for three times after stirred for 12 h under dark environment, the precipitation was collected for further use. To seal the mesoporous structure, the precipitate was redispersed into 18 mL of deionized water, 2 mL of ethanol, and 50 μL of TEOS After reaction for 15 min, the UCNP@mSiO_2_–ICG nanoparticles with sealed structure were obtained.

### *In vitro* biocompatibility based on MTT assay

Standard MTT assay was carried out using L929 cell lines. Firstly, L929 cells were put in a 96-well plate (about 6000 per well) to obtain monolayer, and then material was added with gradient concentrations (1000, 500, 250, 125, 62.5, 31.25, and 15.63 μg mL^–1^). As the control group, the blank was added with pure culture. After being incubated for another 24 h, 20 μL of MTT solution was added to each well, and all the wells were incubated for another 4 h. Then, the mixture was discarded, and DMSO (150 μL) solvent was added to dissolve the produced formazan. Finally, the plate was put on the micro-plate reader, and the absorbance values were recorded at the wavelength of 490 nm.

### *In vitro* biocompatibility based on optical microscopy images

The L929 cells were seeded in a 6-well culture plate and incubated with UCNP@mSiO_2_-ICG for 24 h. Then, the cells were rinsed with PBS three times, and fixed with 2.5% formaldehyde (1 mL per well) for 10 min, and rinsed again with PBS three times. The nuclei were dyed with DAPI solution (20 mgmL_1 in PBS, 1 mL per well) for 10 min for labeling the nucleus. After that, the cells were rinsed with PBS three times.

### Phantom experiment of pure ICG and UCNP@mSiO_2_-ICG

Experimental validation was first performed by imaging nanoparticles filled in a 1.5-mm inner diameter silicon tube. For the control experiment, the tube was filled with ICG. PA excitation (800 nm) was provided by an Nd:YAG pumped optical parametric oscillator (OPO) laser (Surelite^TM^ OPO Plus, Continuum) with 10 Hz pulse repetition rate and 10 ns pulse duration. To increase the sample’s photobleaching rate, we also illuminated both phantoms with CW 808 nm light from a laser diode (L808P1000MM, Thorlabs). The pulsed and CW light beams were combined by a bifurcated fiber bundle with two circular inputs and one circular output. The maximum light intensity at the imaging plane was around 18 mJ/cm^2^ for the pulsed light, and 400 mW/cm^2^ for the CW light. The intensity of either light was below the American National Standards Institute (ANSI) limit for pulsed 800 nm (32 mJ/cm^2^) and CW 808 nm (3.29 W/cm^2^), respectively. PA signals were acquired by a 128-element clinical linear transducer array (ATL/Philips L7–4) with 5 MHz central frequency. Signals from the transducer array were further amplified by 54 dB and digitized by a 128-channel ultrasound data acquisition system (Vantage, Verasonics) with a 20 MHz sampling rate. The raw channel data was reconstructed using the universal back-projection algorithm^49^. During the experiment, both phantoms were exposed to the CW laser for 10 mins, and PA images were acquired at the beginning and the end of the 10 mins exposure.

### Depth imaging in chicken breast tissue

To identify the maximum imaging depth, we imaged a tube of UCNP@mSiO_2_-ICG embedded in a chicken breast tissue. The experimental procedure is similar to the one mentioned in reference^46^, which holds the current world record for PACT imaging depth (11.6 cm). The tube phantom was made of a 5-mm-inner-diameter Tygon tube filled with UCNP@mSiO_2_-ICG (concentration of UCNP@mSiO_2_-ICG is1 mg/mL, the loaded amount of ICG is lower than 10/100 w/w)_._ The experiment was performed in a 500 mL beaker, whose bottom was covered with 8-cm-thickness chicken breast tissues. The tube was placed on the top of the tissue, and we gradually stacked chicken breast tissues on the top of the tube. The 800 nm pulsed laser was routed to the top surface of chicken breast tissue through a 1.2 cm diameter cylindrical fiber bundle with approximately 50% coupling efficiency. The light intensity over the 1.5-cm-diameter illuminated region was around 25 mJ/cm^2^ ^50^. The PA signal from the tube was detected by the ATL/Philips L7-4 transducer and digitized by the Vantage system. For comparison, we also imaged a tube of ICG, using the same imaging setup.

### Animal experiment of pure ICG and UCNP@mSiO_2_-ICG

All the animal experiments were performed in compliance with the animal protocol approved by Institutional Animal Care and Use Committee at University at Buffalo. Light illumination conditions in the animal experiment were the same as for the phantom experiments. Two ND4 Swiss Webster mice were imaged immediately after a tail vein injection of 200 μL UCNP@mSiO_2_-ICG and 200 μL ICG, respectively. During the experiment, each mouse was exposed to 808 nm CW light and imaged continuously with pulsed light (800 nm) for 70 seconds. PA signals were detected by a custom-made three-quarter transducer array with 128 elements and 5 MHz central frequency. The radius of the ring array was 40 mm and each element formed an elevation focus at 35 mm. Thus, elevation resolution and receiving sensitivity are relatively uniform at the central 10 mm radius region. The same Vantage system was used for signal acquisition.

### Data availability statement

The datasets generated during and/or analysed during the current study are available from the corresponding author on reasonable request.

## Electronic supplementary material

Supplementary information
