# Supplementary material for: Stable ICG-loaded upconversion nanoparticles: silica core/shell theranostic nanoplatform for dual-modal upconversion and photoacoustic imaging together with photothermal therapy
Source: Sci Rep. 2017 Nov 16;7:15753. doi: 10.1038/s41598-017-16016-x (PMC5691150; doi:10.1038/s41598-017-16016-x)
Supplement: Supplementary file 1 — Supplementary information [file 41598_2017_16016_MOESM1_ESM.docx]

**Supporting Information**

Stable ICG-loaded upconversion nanoparticles: silica core/shell theranostic nanoplatform for dual-modal upconversion and photoacoustic imaging together with photothermal therapy

Ruichan Lv, Depeng Wang, Liyang Xiao, Guanying Chen, Jun Xia, and Paras N. Prasad


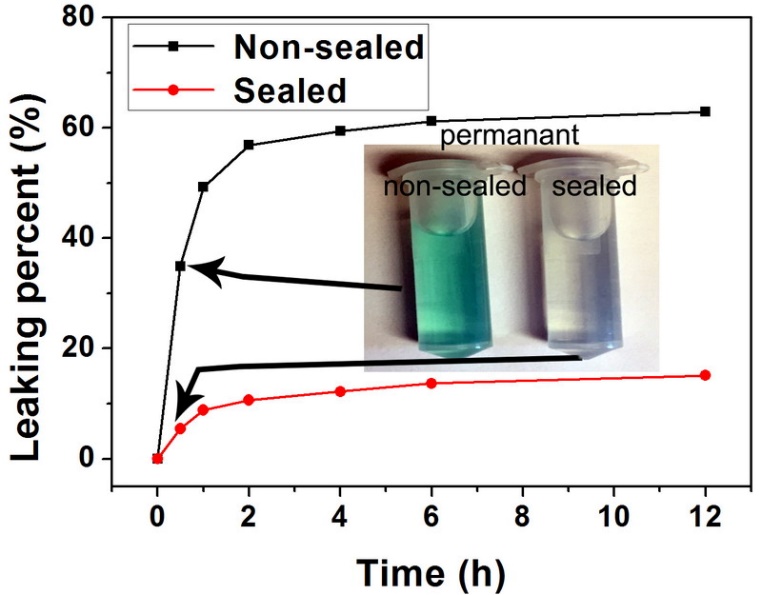


**Figure S1** The leaking properties of non-sealed and sealed UCNP@SiO_2_.


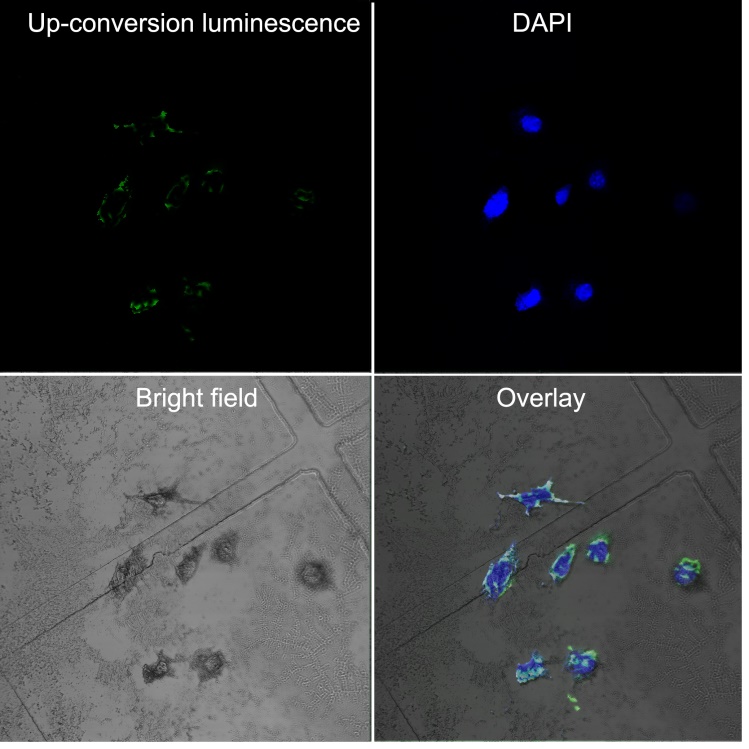


**Figure S2** Inverted fluorescence microscope images of HeLa cells incubated with UCNP@mSiO_2_-ICG upon 808 nm NIR light irradiation.


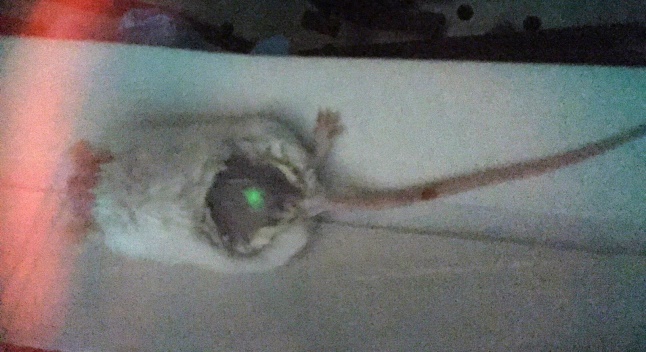


**Figure S3** Photograph of the upconversion luminescence under 800 nm irradiation after subcutaneous injection of the nanoparticles.


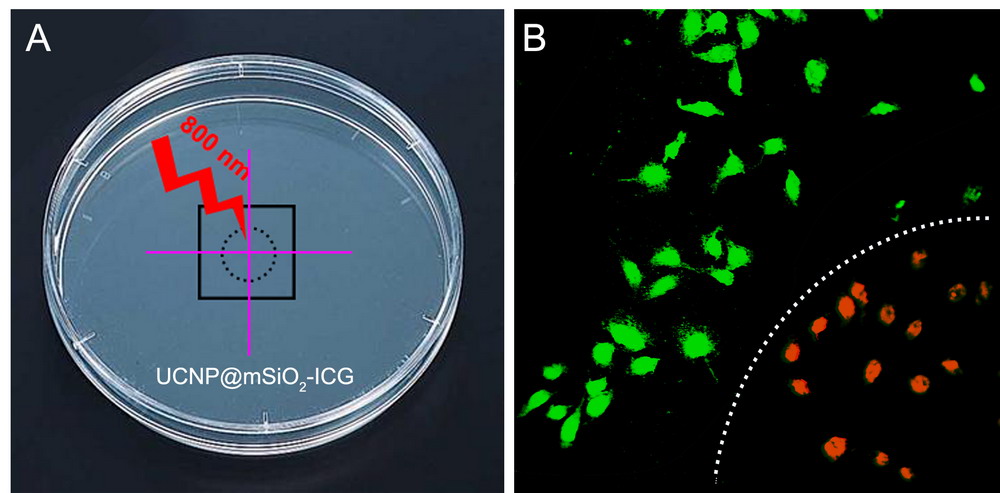


**Figure S4** (A) The scheme of photothermal experiment.(B) The final confocal laser scanning microscopy image of UCNP@mSiO_2_-ICG with (within the circle) and without (outside the circle) 800 nm irradiation.


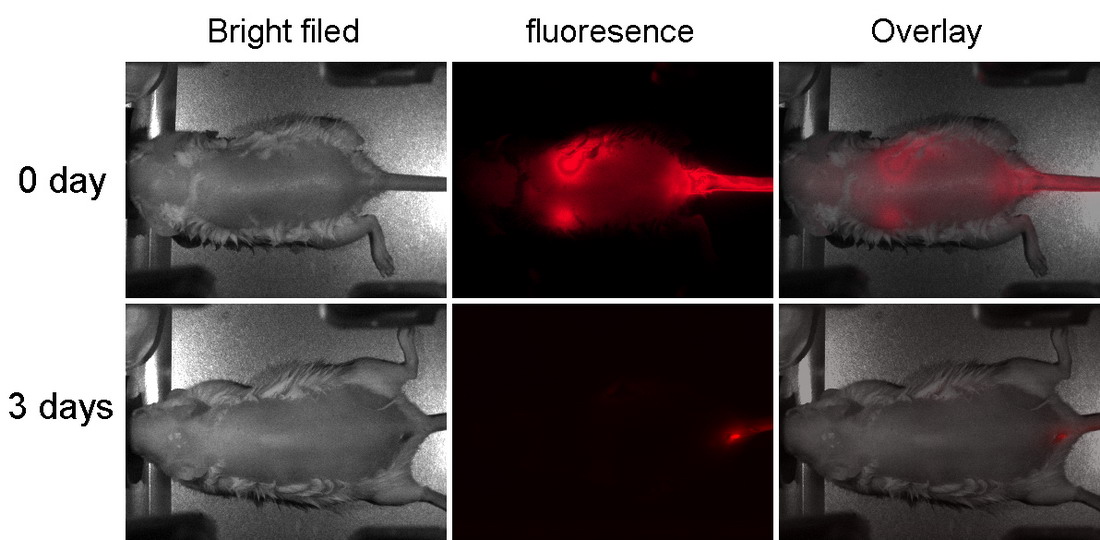


**Figure S5** *In vivo* distributions of nanoparticles after intravenous injection. Images were acquired immediately after the injection and at day 3.


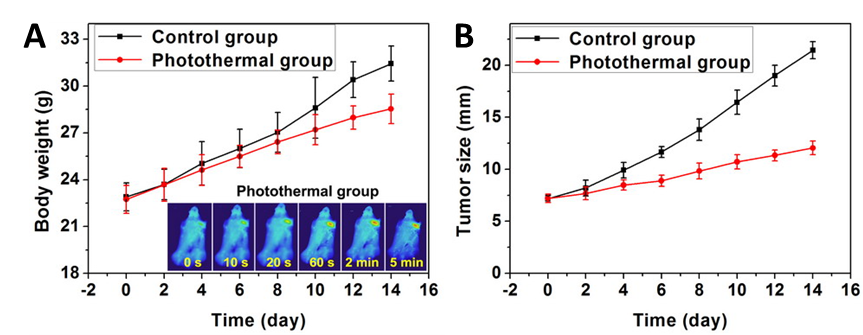


**Figure S6.** *In vivo* photothermal experiments. (A) Body weight of the control group and the photothermal group. Inset is the infrared thermal images of mice (with UCNP@mSiO_2_-ICG injected intratumorally) under 808 nm irradiation. (B) Tumor size change for control and photothermal groups.
